# Adapting the deliberative democracy approach to LMIC settings: a case study in Nigeria

**DOI:** 10.3389/fpubh.2025.1613903

**Published:** 2025-10-17

**Authors:** Laura M. Gaydos, Kabiru Salami, Wasiu Yusuf, Ifeoma Idigbe, Olutosin Awolude, Olabanjo Ogunsola, Tyree Staple, Priscilla Ezemelue, Oluseye Ajayi, Oliver Ezechi, Colleen M McBride, Lisa Flowers

**Affiliations:** ^1^Department of Health Policy and Management, Emory University, Atlanta, GA, United States; ^2^Department of Sociology (Medical Sociology), University of Ibadan, Ibadan, Nigeria; ^3^Clinical Sciences Department, Nigerian Institute of Medical Research, Lagos, Nigeria; ^4^Obstetrics and Gynecology Department, College of Medicine, University of Ibadan, Ibadan, Nigeria; ^5^APIN Public Health Initiatives, Abuja, FCT, Nigeria; ^6^Department of Gynecology and Obstetrics, Emory University, Atlanta, GA, United States; ^7^Department of Behavioral, Social and Health Education Sciences, Emory University, Atlanta, GA, United States

**Keywords:** deliberative democracy, LMIC, HIV, cervical cancer, HPV, women living with HIV

## Abstract

**Background:**

Developing sustainable health promotion interventions in low- and middle-income countries (LMICs) faces challenges due to limited infrastructure and diverse cultural contexts. Community engagement is essential for effective health promotion, but higher intensity strategies may be infeasible in under-resourced settings. This study aimed to adapt the Mentor Mother (MM) HIV peer program to include HPV self-screening for Nigerian women living with HIV using deliberative democracy (DD) principles.

**Methods:**

The study utilized a tiered DD approach to explore stakeholders’ perspectives on feasible and sustainable strategies for the MM program. The process included two tiers: an initial deliberation among the research team and a subsequent community deliberation. The research team deliberation involved online sessions to identify feasible program adaptations as well as a model deliberation process. The community deliberation included a diverse group of stakeholders who participated in a two-day conference, engaging in small and large group discussions to reach consensus on program adaptations.

**Results:**

The research team identified two options for HPV sample collection and result delivery. The community deliberation reached consensus on both questions. For sample collection, the preferred option was for MMs to educate women in organized groups and transport samples to the laboratory. For result delivery, the consensus was for MMs to return all results to patients after additional training. The process demonstrated high levels of participant satisfaction, increased self-efficacy in explaining HPV screening, and adherence to DD principles of inclusivity, reasoned justification, and societal perspective.

**Conclusion:**

The DD process was feasible and effective in adapting the MM program for HPV screening in Nigeria. The approach empowered community members and enhanced the intervention’s development. However, adaptations were necessary to address cultural norms and logistical challenges. The study highlights the potential of DD to inform health promotion strategies in LMICs, ensuring interventions are culturally appropriate and sustainable.

## 1 Introduction

Developing sustainable population-based health promotion interventions in low and middle-income countries (LMICs) faces numerous challenges. The infrastructure to sustain effective interventions is often lacking and rural communities are widely dispersed in ways that impede dissemination of these programs (1, 2). LMIC settings are also culturally, linguistically, and religiously diverse. Moreover, many health promotion endeavors are developed in partnership with non-government organizations (NGOs) funded by high-income countries with variable cultural literacy. Effective health promotion strategies in LMICs must therefore be context-specific, community-informed, and responsive to local realities (3, 4).

Community-based approaches that center peer engagement—such as the Mentor Mother (MM) model—have demonstrated success in improving health outcomes among women living with HIV (WLWH) (5, 6). Integrating such models with services like cervical cancer (CC) screening is particularly important for WLWH, who face elevated risks of persistent Human Papilloma Virus (HPV) infection and CC (7, 8). The World Health Organization (WHO) now recommends targeted screening and prevention strategies, including self-collection for HPV testing, which is both acceptable and feasible in many LMIC settings (9, 10). Nonetheless, barriers such as stigma, limited infrastructure, and fragmented services continue to hinder integration (11, 12). Despite the high burden of CC, HPV screening coverage in Nigeria remains low, with studies reporting rates as low as 8.3% among eligible women (13).

Expanding access to HPV screening for WLWH is essential not only from a clinical standpoint but also from a health systems and equity perspective. The MM program presents a scalable and trusted platform for health communication and service delivery in Nigeria. MMs—trained HIV-positive peers—have built strong, trust-based relationships with the communities they serve, and their integration into CC screening efforts offers a pathway to increase uptake, ensure follow-through, and bridge service delivery gaps in under-resourced settings. Leveraging this model to include HPV screening capitalizes on existing infrastructure while aligning with national and global goals to reduce CC morbidity and mortality among high-risk populations.

In this context, community engagement strategies are considered standard practice if health promotion programs are to be effective and sustainable. Engagement strategies span a continuum, from lower-intensity forms like informing and consulting communities, to higher-intensity forms like community co-ownership (14, 15). When health promotion collaborations include partners from high-income and LMIC settings, differences in cultural literacy and positionality underscore the need for more inclusive, participatory approaches to intervention design (16).

Deliberative democracy (DD) is a promising high-intensity engagement framework that emphasizes structured dialog, the consideration of expert testimony, and inclusive participation to promote reasoned consensus (17, 18). DD has gained traction in LMICs as a method to ensure legitimacy and community ownership in public health programs, with successful applications in priority-setting for HIV programs in Indonesia (19), cancer prevention in South Africa (20), and noncommunicable disease control in Kenya (21). DD methods also enable marginalized groups to express their preferences and share experiential knowledge, potentially offsetting historic patterns of exclusion in global health research (22).

Our large international and interdisciplinary team considered options for higher intensity engagement strategies to inform development of a sustainable program to increase access to HPV screening among WLWH. The research team comprised local NGOs, in-country academic institutions, and U. S.-based researchers. The goal was to adapt an existing HIV peer support program known as Mentor Mothers shown previously to be effective (5, 6, 23, 24), to add HPV self-screening. Despite prior evidence supporting both peer-led and self-sampling approaches, few studies have examined how deliberative methods might inform program adaptations for WLWH in LMICs.

To this end, we decided to undertake a stakeholder engagement process based on principles of DD (17, 25). DD is a structured deliberation process based on the assumption that priority setting is a value-based process (26).

It brings together a diverse group of stakeholders—including those directly affected by a health issue—and equips them with balanced, factual information to enable reasoned dialog. Participants deliberate on competing values, weigh trade-offs, and work toward consensus solutions that are thought to promote the common good (27). Deliberators are provided with neutral factual information about the issue via “expert testimonies,” encouraging them to air differing viewpoints, interests, and experiences in small and large group discussions. The deliberators then deliberate about the tradeoffs they view to be important to come to a consensus that, in theory, would maximize the common good. DD has been used in several international/LMIC settings for a number of different health and policy topics including noncommunicable disease control in Kenya (21), HIV control in Indonesia (19) and health priority setting (22, 28, 29) and heritable human genome editing in South Africa (20).

Arguably, this value-based process could be especially beneficial for understanding and sustaining health promotion programs in LMICs that have experienced the disempowering effects of historical colonialism (30). Previous literature has found that DD methods provide more authentic public opinions (16, 18, 31, 32). Enlisting communities in LMICs to generate and thoughtfully consider potential pros and cons of health policies and programs through the lens of personally experienced disparities can be an act of empowerment (16). This focus on procedural fairness enables transparency in the decision-making process and how consensus is established, which are thought to increase the legitimacy of final decisions (33).

Informed by previous research (16, 28), we considered three key democratic principles that would suggest a quality deliberation process: *Consideration of Balanced and Factual Information:* DD requires that participants have basic and unbiased understanding of the issues and tradeoffs to enable active discussion of the questions being deliberated. *Inclusivity:* The deliberation group should reflect on the diversity of citizen and consumer views and life experiences. Deliberation cannot be fully democratic if participants do not have the opportunity to contribute. *Deliberation:* participants discuss and weigh differing, and often competing, social values to reach consensus as a group (27). Members must have equal opportunity to take part in the discussion and deliberate, which involves listening and reflecting on others’ perspectives before reaching conclusions. Members are encouraged to adopt a societal perspective on the issue in question, where the deliberation focuses on what is best for society, rather than on what is best for individual participants. In addition, the group reflects on what they hear and provides their rationale when offering comments.

In our tiered DD process, we aimed to identify what the research team regarded as MM program adaptations that would be feasible and have the greatest potential to be sustainable in these geographically dispersed settings. In the second step, we presented these options in the form of deliberation questions and expert testimonies to assist deliberators to decide on which options to pursue. In this report, we describe our findings relating to the feasibility of conducting a quality deliberation process in an LMIC setting to arrive at our intervention adaptations.

## 2 Materials and methods

### 2.1 Overview

The DD approach was utilized to explore stakeholders’ perspectives on feasible and sustainable strategies for adopting the MM program to increase access to HPV screening among Nigerian WLWH.

DD is generally conducted as a single deliberation conference that includes stakeholders and citizens representing multiple constituencies and perspectives. Because this activity was to be conducted by study collaborators encompassing a new collaboration with a large interdisciplinary and multi-cultural team who were unfamiliar with the DD methods, we elected to conduct an initial deliberation among the research team.

**Figure 1:**
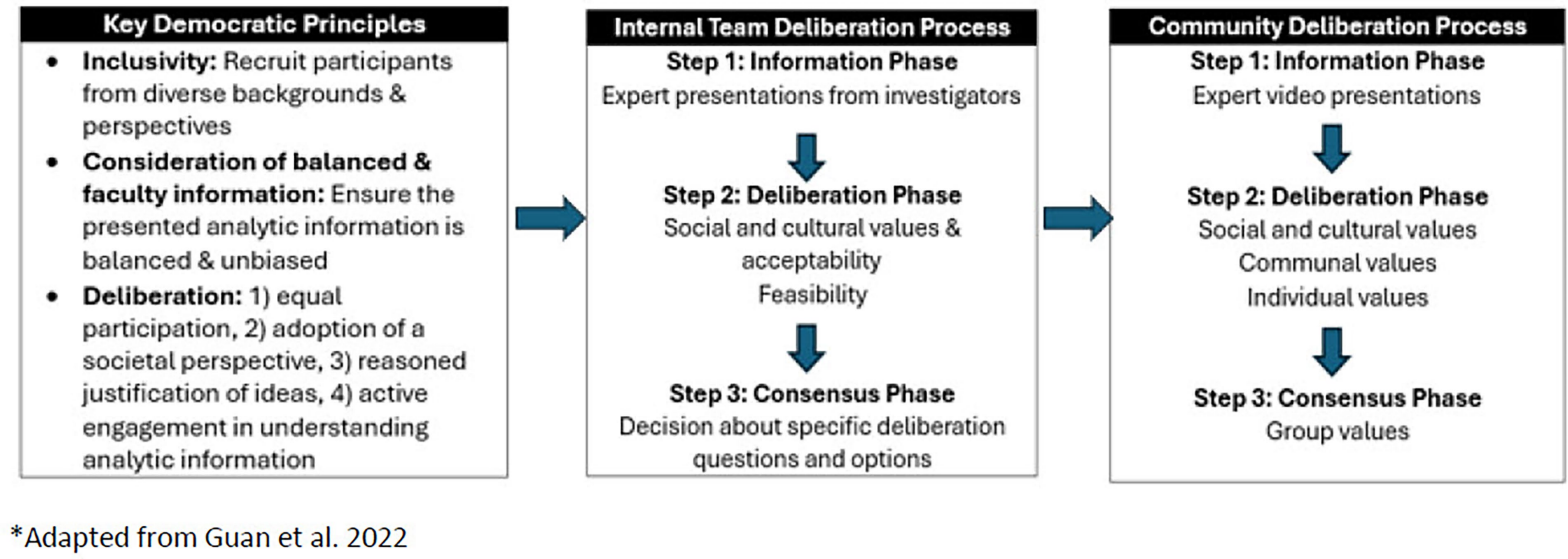
Adaptation of deliberative democracy approach.

Figure 1 shows the addition of the tier 1 research team deliberation to standard DD methods.

### 2.2 Positionality statement

Prior to commencing our research team deliberation, it was incumbent upon our team to acknowledge our positionality. Our research team comprises collaborators from an American university, a Nigerian university, a Nigerian research institute, and a Nigerian non-governmental organization (NGO). As a transnational, interdisciplinary group, we recognize that our varied institutional affiliations, professional roles, and personal identities shape our approach to research and community engagement.

Team members based at a large academic medical center and university in the United States bring expertise in DD, public health policy, gynecology, and behavioral sciences. The American collaborators acknowledge that our positioning within a high-income country and Western academic framework confers privileges that may influence research priorities, access to funding, and the interpretation of data. We remain conscious of the potential for power imbalances in global health collaborations and are committed to practices that promote equity, shared leadership, and local relevance.

Our Nigerian collaborators—clinicians, social scientists, and public health experts—are deeply embedded in the healthcare and sociocultural contexts of the communities we study. Their longstanding work with WLWH, including through the MM program, provides the critical on-the-ground perspective needed to ensure that our research is culturally grounded, ethically sound, and practically feasible. Their contributions were central to adapting the DD framework to the Nigerian context and to guiding community-engaged methodologies that respected local norms and priorities.

We acknowledge the ongoing structural inequities in global health research and therefore, intentionally engaged in a co-learning process, including internal deliberation sessions among the research team, to reflect on our positionalities and collectively shape study design and implementation. Our team aimed to create a research process that was collaborative, respectful, and responsive to community voices, especially those of WLWH, whose lived experiences are central to our study.

### 2.3 Tier 1: research team deliberation

In the first 6 months of the funding period, the principal investigators and co-investigators on the project engaged in 24 h of online sessions that mimicked the planned deliberation process to be conducted with community constituencies. The research team deliberation was facilitated by a co-Investigator with expertise in DD (CM) and comprised eight investigators with expertise that included: DD methods, implementation science, CC prevention and treatment, and HIV/AIDS prevention and treatment. The research team deliberation was guided by the overarching question, *what adaptations to the Mentor Mother program would be feasible and sustainable in the target settings that could be prioritized by recruited deliberators?* We then decided on two more specific deliberation questions driven by areas for which our own research team was not in agreement: Question (1) *What is the approach for collecting HPV samples for women who are HIV+ that best balances benefits and challenges?* And Question (2) *What is the approach for returning results to women who are screened for HPV that best balances the benefits and challenges?*

Investigator-led, co-learning presentations were used as expert testimonies to give the research team a shared understanding of the pros and cons of different program adaptations for the sustainability of the adapted MM intervention. Expert testimony topics included: DD methodology, CC and HPV screening, steps involved in self-collection of biological samples, and the MM program. Questions were addressed and discussions initiated among the research team.

After the presentations, researchers identified the pros and cons of different locations for MM’s to educate women and to conduct self-testing. A second deliberation followed a similar process for discussing the role of MM in delivering HPV screening results. Research team members broke into small groups as would be done in a formal deliberation, discussed the pros and cons of possible approaches, came to consensus and presented their rationale for the approach they favored to the larger team for further discussion.

The team deliberations resulted in two options for each of the deliberation questions. For question 1: *What is the approach for collecting HPV samples for women who are HIV+ that best balances benefits and challenges?* our team developed two options: the first option was for MMs to visit women in their homes to educate and facilitate home-based self-collection and for women to transport their own samples to local clinics for analysis. The second option was for MMs to educate women as a group about CC self-screening and provide screening kits in designated meeting locations with MMs transporting samples back to the laboratory or clinic.

For question 2: *What is the approach for returning results to women who are screened for HPV that best balances the benefits and challenges?* the first option was for MMs to notify women of normal/negative results with local nurses contacting women who had positive screening results. The second option was to provide MMs with additional training in how to return all results (both positive and negative) to patients. The final deliberation questions and options that were agreed upon after the team deliberation represented areas in which the experts on our research team held disagreements about the best approach for the intervention.

### 2.4 Tier 2: stakeholder deliberations for the community

#### 2.4.1 Recruitment for the community deliberation

The research team developed a recruitment rubric intended to maximize the likelihood that a diverse group of stakeholders and members of the general public would be represented at the deliberation conference. Additionally, the team sought to include a diverse group of participants based on demographic factors including gender, age, and state (with a balance from the northern and southern regions of the country) (see Table 4 for diversity of actual deliberation participants). The research team developed a comprehensive list of potential deliberators that included representatives from NGOs associated with the MMs program, seasoned mentor mothers, WLWH, and nurses in community clinics. Community members not affected by HIV also were suggested as representatives of the general public. Ten key perspectives were identified as shown in Table 1.

**Table 1:** Perspectives sought for deliberation.

Perspective	Justification for inclusion
Association of women living with HIV/AIDS in Nigeria (ASHWAN)	Nationally representative group advocating for WLWH
Clinic nurse managers	Will oversee intervention in clinic sites
Community members (not person living with HIV)	Outside perspective from someone not directly related to care for WLWH
Gynecologists	Will evaluate and treat WLWH found to have high-risk HPV
Husband or male family members of woman living with HIV	In many parts of Nigeria (especially the North) male family members determine behaviors of women
Journalists against AIDS	Advocate for the rights and wellbeing of people living with HIV
MMs in HIV program	MMs will be implementing the intervention
Laboratory coordinators (National/Regional)	Laboratory will be an important part of the intervention to ensure that samples are received and results are returned
Nurse/nurse counselors	Nurses currently return most HIV results
WLWH	The target population for the intervention

Additionally, the goal was to include deliberators who would feel confident to express the views of their constituency in small and large group discussions. Based on these considerations and the research team’s brainstorming, a list of 21 people was generated. A letter to each person was sent from one of our collaborating non-governmental institutions in Nigeria inviting them to participate in a two-day conference at the Nigerian Institute of Medical Research in Lagos, Nigeria.

#### 2.4.2 Materials development

Our team identified key content that would enable a shared understanding of issues related to HPV screening that would enable community participants to deliberate on our questions. The objective was to be as parsimonious as possible in identifying content as the expert testimonies had to be relatively short, if we were to contain the process to a two-day deliberation. We generated a list of 8 topics we considered essential background information and developed draft slide decks for each topic; slide decks were reviewed by the full research team and finalized. Each slide deck was turned into a video recording with a voiceover recorded by a Nigerian voice actor in English, which was selected given that all of the deliberation participants were expected to have moderate to good verbal English fluency. Video topics are shown in Table 2. We also adapted in-depth workbooks from Guan et al.’s previous study (5) for the deliberators to use during the conference and a similar guide for deliberation facilitators to use during the conference. The workbooks were reviewed by Nigerian colleagues for cultural awareness and piloted with a group of three MMs who provided feedback and allowed us to revise them to be most useful and acceptable for the intervention.

**Table 2:** Community stakeholder deliberation video topics.

Community stakeholder deliberation video topics	Video length
Video 1: Introduction to the citizen deliberation process	6 min
Video 2: Understanding population screening	4 min
Video 3: Interaction between HIV, HPV and CC	3 min
Video 4: HPV screening	3 min
Video 5: Self-sampling for HPV screening	3 min
Video 6: MMs and their role serving women living with HIV (WLWH)	3 min
Video 7: Understanding HPV test results	2 min
Video 8: Options for who should return HPV for WLWH	4 min

#### 2.4.3 Training of deliberation facilitators and scribes

Five Nigerian facilitators were recruited for the deliberations. The facilitators were experienced focus group facilitators who had worked with and were recruited by Nigerian research partners. Members of the team with expertise in DD conducted 4 h of facilitation training (two, two-hour virtual sessions) in the weeks prior to the deliberation conference. The training consisted of mock deliberation sessions and walking through the facilitator manual in detail. We also conducted a “mop up” training the night before the deliberation conference in which two members of the research team reviewed the protocols, role played their specific parts and clarified any outstanding questions. In addition to the facilitators, four scribes (also women who had assisted our NGO partners in previous qualitative studies) participated in the “mop up” training during which they role played their roles in a mock deliberation. Logistic roles such as audio recording also were practiced during this session.

#### 2.4.4 Deliberation conference procedures

Our Deliberation conference consisted of a mix of educational sessions, small group and large group deliberations as shown in Table 3.

**Table 3:** Sequence and content of the DD conference.

Day 1	
1st plenary session (2 h)	Overview and introductions Overview of the CHESS project Video presentation of the DD process and how it will direct the study implementation (Expert testimony #1)
Tea break (30 min)	
1st small group deliberations (45 min)	Mock deliberation and voting on humorous topic
2nd plenary session (80 min)	View expert testimony background videos Understanding population screening (Expert testimony #2) HIV, HPV and CC (Expert testimony #3) HPV screening (Expert testimony #4) Q&A about the content in the videos Watch more expert testimony background videos Self-Sampling for HPV Screening (Expert testimony #5) Mentor mothers and their role with WLWH (Expert testimony #6) Q&A about the content in the videos Introduce deliberation question for day 1
2nd small group deliberations (45 min)	Deliberation and voting on best approach for collecting HrHPV samples from WLWH
Lunch (1 h)	
1st large group deliberation (75 min)	Breakout groups present deliberation pros and cons list to full group Vote on deliberation question 1
Facilitator team debrief (30 min)	Facilitator team debrief with study investigators

Day 2	
3rd plenary session (90 min)	Watch expert testimony background videos Understanding HPV test results (Expert testimony #7) Considerations for who should return HPV results for WLWH (Expert testimony # 8) Q&A about the content in the videos Introduce deliberation question for day 2
3rd small group deliberations (75 min)	Deliberation and voting on best approach for returning HPV test results to WLWH
Lunch (1 h)	
2nd large group deliberation (75 min)	Breakout groups present deliberation pros and cons list to full group Vote on deliberation question 2
Survey, evaluation and wrap up (30 min)	Participants complete evaluation surveys

Day 1, deliberators checked in and completed a baseline survey assessing their knowledge of CC, self-screening, and HIV. Deliberators started with a mock deliberation on a humorous topic (i.e., choice of which of two silly hats to wear on a first date) to understand how the deliberation process worked as well as the consensus voting. In the morning and afternoon sessions, deliberators were asked to watch the expert testimony videos relevant to the day’s deliberation question. The Day 1 question was*: What is the approach for collecting HPV samples for women who are HIV+ that best balances benefits and challenges?* After viewing the expert testimonies and offering an opportunity for follow-up questions, the participants were grouped into four heterogeneous (by role/perspective/geography) breakout groups of 5–6 participants each. Initially, prior to any other discussion, each breakout group facilitator asked the deliberators to individually state their initial vote on the two presented options. Once these initial perspectives were shared, the deliberators worked in their small group to generate a comprehensive list of pros and cons for each option. After the pros/cons list was generated and the group agreed that the list was comprehensive, another round of voting was held in which each participant again voted and explained their rationale for the vote. Voting continued until an 80% consensus on the best option was reached. Once the small groups had achieved consensus, the deliberators reconvened as a large group. Each small group selected a representative to share their list of pros and cons, the results of their small group vote and the general rationale for the consensus. Small group representatives were asked to highlight points of agreement and disagreement and how they were able to reach (or not reach) consensus. At the end of the small group representative presentations, all participants were asked to take a large group vote on the two options. If 80% consensus was not reached, the group would discuss further and vote again.

The same process of small group discussions followed by large group deliberation was repeated for day 2 of the conference. For day 2, the deliberation question was: *What is the approach for returning results to women who are screened for HPV that best balances the benefits and challenges?* Upon completion of day 2 deliberation activities, the participants took the post-survey. This study has been approved by the Institutional Review Boards of Emory University (STUDY00004817), APIN Public Health Initiatives, the Nigerian Institute of Medical Research, and the University of Ibadan. All study participants will be consented to participate.

#### 2.4.5 Data collection

Quantitative data collection included consensus vote tallies at each stage of the deliberation and pre- and post-conference surveys to assess participant knowledge, attitudes and beliefs related to CC prevention for WLWH. We also assessed self-efficacy with questions about perceived confidence in the ability to explain to members of their community about various topics including HIV, CCrisk, and CC screening as well as statements about the importance of their views in informing government decision makers regarding CC prevention. The post survey also included satisfaction questions (e.g., do you feel that you were respected by your group members and how helpful they found each component of the process) and recommendations for the conference itself.

Qualitative data collection included audio recording and transcribing all conference sessions (small group and large group) verbatim. We also collected the pros and cons lists from each of the deliberators. No specific guides were used for the deliberation data collection beyond the presentation of two alternatives and the question of which one best balanced the strengths and challenges. This is consistent with standard DD implementation during which the participants generate the pros, cons, comments and questions rather than responding interview or focus group prompts.

#### 2.4.6 Quantitative data analysis

Quantitative data analysis of survey data was conducted using SPSS (26) to characterize the participants’ demographics, ratings of their experience with deliberation and changes to self-efficacy. We compared pre and post survey scores for each individual as well as comparing pre and post means and significance testing for the group of all participants.

#### 2.4.7 Qualitative analysis

We coded deliberation session transcripts for the following indicators of process quality (5, 27) *speaking opportunities (inclusivity), adoption of a societal perspective on the issues in question (societal perspective) and reasoned justification of ideas (consideration of balanced and factual information)*. With regards to *speaking opportunities*, we measured deliberation participation in terms of number of statements made in each small group and percentage of the comments made by each individual (using the total number of statements made by all participants as part of the same small deliberation group as the denominator). Notably, one set of facilitators (impacting 1 group each day) did not use names during the deliberation, making it impossible for us to count the participation for those groups. *Adoption of a societal perspective* was demonstrated when a participant made a statement related to the greater good/harm of an option on the population at large. *Reasoned Justification* of ideas was demonstrated when a participant used the expert testimony provided or considered the pros and cons of an option.

Coding of deliberation transcripts was completed using MAXQDA software (28). We adapted a qualitative coding scheme used by others to examine the deliberation process (27, 29). One study team member reviewed all transcripts and compared them to the audio recordings. Two study team members coded each transcript. Following initial independent coding, the two coders met with a larger group of investigators to review coding and discuss discrepancies. Additionally, the larger coding group compared themes in the initial coding to the comprehensive list of pros and cons, adding new themes and generating recommendations for adapting the CHESS Mentor Mother intervention.

## 3 Results

### 3.1 Community deliberators

Of the 10 perspectives prioritized in our recruitment rubric, we were able to capture 9 of the perspectives from 21 participants who varied in gender (70% female, 30% male), age, and state (the 8 states in which our study has clinic sites were all represented) as shown in Table 4 above. All 21 invitees responded to the invitation with 19 agreeing to attend and two requesting to send a proxy in their stead. All 21 scheduled participants showed up to day one and day two of the deliberation conference.

**Table 4:** Demographics of community deliberation participants.

Region	State	Gender	Age	Perspective
Central	Abuja	Male	40s	Regional or national coordinator national sample referral network (Laboratory)
Central	Abuja	Female	30s	Association of women living HIV/AIDS in Nigeria (ASHWAN)
North central	Plateau	Female	50s	Clinic nurse manager
North central	Jos	Male	40s	Community member (non person living with HIV/AIDS)
North central	Plateau	Male	40s	Gynecologist
North central	Plateau	Female	40s	Nurse
North central	Plateau	Female	40s	Nurse/Nurse counselor
Northern	Kano	Male	50s	Gynecologist
Northern	Kano	Female	40s	Nurse/Nurse counselor
Northern	Kano	Female	40s	Woman living with HIV/ Mentor Mother
Southeastern	Anambra	Female	50s	Gynecologist
Southeastern	Anambra	Female	40s	Nurse/Nurse counselor
Southeastern	Anambra	Female	50s	Woman living with HIV/ Mentor Mother
Southern	Delta	Male	50s	Gynecologist
Southern	Delta	Female	30s	Nurse/Nurse counselor
Southern	Delta	Female	40s	Woman living with HIV/Mentor mother
Southwestern	Oyo	Female	40s	Woman living with HIV/Mentor mother
Southwestern	Oyo	Female	50s	Clinic nurse manager
Southwestern	Oyo	Female	40s	Gynecologist
Southwestern	Lagos	Female	Unknown	Journalist against AIDS

### 3.2 Satisfaction with the deliberation process

All 21 deliberators (100% completion rate) rated their satisfaction with the process to be very high (Table 5). Ratings were particularly high for feeling respected by the group and feeling heard by the facilitator.

**Table 5:** Respondent satisfaction with the community deliberation (1–10 scale).

Question	Mean value	Range
Do you feel that your opinions were respected by your group members?	9.7	8–10
Do you feel you were listened to by your facilitator?	9.7	9–10
Do you feel that the process that led to your group’s final vote was fair?	9.2	4–10
How willing were you to abide by the group’s final vote, even if you personally have a different view?	8.8	6–10
How much did attending the session change your understanding about CC screening in Nigeria?	9.3	3–10
How much did attending the session change your opinion about CC screening in Nigeria?	8.9	1–10

### 3.3 Self-efficacy related to HPV screening knowledge

Table 6 shows pre- to post-deliberation changes in self-efficacy to explain to community members the concepts covered in the expert testimonies. Confidence (1-low to 5-high) to explain increased for all measures and this increase was statistically significant for 5 of the 9 measures. Increases were highest for deliberators with respect to explaining HPV self-testing to women with HIV, what a “high-risk” result means, and how often women need CC screening. Moreover, there were significant increases in indicators of feeling empowered by the process as indicated by increases in perceptions that “Nigeria’s health policy makers want to hear my opinion about how CC screening should be conducted.”

**Table 6:** Respondent self-efficacy related to HPV screening.

Question	Pre-deliberation		Post- deliberation		
	Mean	SD	Mean	SD	Significance
I am confident I can explain to members of my community how HPV influences CC risk	4.20	1.056	4.70	0.571	0.076
I am confident I can explain to members of my community how women can collect their own sample to be tested for HPV	3.84	1.119	4.58	0.507	0.007*
I am confident I can explain to members of my community what a high-risk CC screening result means	4.05	1.129	4.74	0.452	0.011*
I am confident I can explain to members of my community how often women need to get CC screening	4.10	1.119	4.80	0.410	0.012*
I am confident I can explain to members of my community why WLWH are more likely to get CC	4.42	1.170	4.84	0.375	0.134
My opinions can influence health policy related to CC screening access in Nigeria	1.57	1.076	4.57	0.598	0.000*
Nigeria’s health policy makers want to hear my opinion about how CC screening should be conducted	4.00	0.918	4.50	0.607	0.038*
Community members working together can influence Nigeria’s health policy makers’ views on CC screening	4.33	1.017	4.62	0.590	0.229
My opinions are not important to Nigeria’s health policy making related to CC screening	1.57	1.076	1.71	1.189	0.658

*Significant at α ≤ 0.05.

### 3.4 Quality of the deliberation process

#### 3.4.1 Speaking opportunities (inclusivity)

Participant speaking counts ranged from 1 comment to 63 counts of comments with a mean of 14.6 and range of percentage of participation from 3.5 to 60% of comments made within the small groups. Notably, many of the lower counts of participation included long comments versus shorter, broken up comments for the very high counts.

#### 3.4.2 Reasoned justification of ideas (consideration of balanced and factual information)

The quality of a deliberation is upheld when participants show a willingness to explain the rationale for their viewpoints based on the expert testimonies or the perspectives of other deliberators. In all small group discussions, participants regularly pointed to the expert testimonies to support the pros and cons they suggested. For example, one nurse participant referenced the expert testimony regarding legal requirements for disclosing health data in Nigeria saying *“Even though the video says, I agree that the Nigerian government have approved disclosing HIV by the Mentor mothers. But my argument is that HIV is not the same as HPV. HIV is not a death sentence. It’s not something that leads to death. HPV is a cancer precursor. So it’s a more problem.” (Group 1, Day 2).*

Additionally, deliberators considered the viewpoints of others as they weighed the optimal choice for each question. For example, one gynecologist participating in the deliberation noted a change in his perspective from initially believing that the program should be home-based to understanding the value of the community-based intervention saying: *“The issue is that the key thing I have for option one (home-based) is confidentiality. But listening to the rest of the group, I’m now wondering if the fact that if you put a group together that know themselves that ability to get peer support among them knowing that they all have the same thing - I’m wondering if that will outweigh the potential confidentiality. So, I’m veering toward saying that maybe, maybe it will outweigh the confidentiality because they are able to support themselves.” (Day 1, Group 1).*

### 3.5 Adoption of societal perspective

Adopting a societal perspective was demonstrated when participants shared a perspective that valued the group welfare over their own personal interests. While there were many examples of pros and cons offered during deliberation that focused on the societal perspective, we also noted responses that clearly demonstrated a focus on improving societal welfare over one’s personal views. For example, a participant who was a nurse in the clinics and had previously expressed concerns that nurses were being left out of the study process noted that after the deliberation *“What really made me choose option 2 (Mentor mothers returning all results) … is the PMTCT (prevention of mother to child transmission of HIV). If we all remember the PMTCT program, it went really well. We’ve been working with mentor mothers. We’ve been sharing issues and deliberations together… So, I believe these two, as we are working together with the nurses and Mentor mothers will achieve the best.” (group 2, day 2).* This statement demonstrates that although the participant was concerned about having her role as a nurse valued, she understood that having MMs return all results to the women was better for patients because of the increased level of trust between patients and MMs. Another nurse very specifically commented on the need for societal perspective during deliberation when she stated *“We’re not talking about the material benefit. Let us talk about the benefit of the patient.” (Group 2, day 2)* Similarly, in the heat of deliberation on day 2, a nurse changed her vote to support MMs returning all results saying *“That we did not pick nurses. If I had that impression, I will vote against profession. Let us vote in the interest of our community, in the interest of public health.” (Group 3, Day 2).*

### 3.6 Deliberation day 1 results

Deliberators came to agreement on the first deliberation question: *What is the approach for collecting HPV samples for women who are HIV+ that best balances benefits and challenges?* In small group discussions, there was strong consensus that option two, having MMs provide education to WLWH in organized groups and with MMs transporting samples back to the laboratory or clinic. Multiple reasons were given for selecting the community option, including issues of transportation and peer support.

As one participant noted*: Why I choose option two is because for the client to say to collect the sample by herself to bring it to the facility. She may tell you she do not have transport to come. Transportation. She will tell you that she do not have transportation to come (Group 1, Day 1).*

Another reason given to support the community option was the efficiency of group education. A participant who identified herself as a MM noted: *When you are doing one by one, I’ll be repeating myself. But it will be group like this one is saying organized group. Talk about what CC is, how to go about the testing. At least I am going to give them information at the same time. And all the women, if there is going to be a demonstration of how to use the kit. It will be one off and they will understand the reason why we are doing it. It’s a group thing (Group 3, Day 1).*

Option two was ultimately selected by all four of the breakout groups. Three of the four groups reached 80% consensus in the first round of voting. The fourth group reached 80% consensus on their second round of voting. In the larger group, 100% consensus was reached in the first round of voting.

### 3.7 Deliberation day 2 results

Deliberators took longer to come to agreement on the second question: *What is the approach for returning results to women who are screened for HPV that best balances the benefits and challenges?* With option 1 being return of HrHPV positive results by clinical staff and option 2 being return of all results by MM. Small group discussions did not come to immediate consensus. One participant noted the importance of the trust between patients and Mentor mothers saying: *Patients trust Mentor Mothers more than the nurses because they believe that they are the same group. So when a mentor mother counsels a patient, the patient pays more attention than the nurse. They believe that the nurses are just there wearing white. But the mentor mother wears the same shoes as them. So, I believe they trust mentor mothers* (*Group 2, Day 2*).

There was also considerable discussion about option two to train MMs with extra training to return all results (both positive and negative) to patients.

One participant noted that they had concerns about the ability to train MMs to deliver complex results noting: “*The cons, what, what I have against, the potential problem is that there may be difficulty with training or learning on the part of the mentor mother*.” (*Group 4, Day 2*).

In contrast, another participant stated: *“But the option here says they will be trained. and I feel there’s really nothing rocket science in disclosing, a positive result*.” (*Group 4, Day 2*).

One group came to 80% consensus in the first round of voting; two groups came to consensus on option 2 in two rounds of voting; the final group came to consensus after three rounds of voting. The large group consensus process required three rounds of voting to reach 80% consensus. The first vote showed a score of 38.1% for option 1 and 61.9% for option 2. Similarly, there was no consensus in the second round with option 1 having 23.8%, and option 2 having 76.2%; consensus was not reached until the third round when option 1 got 19% and option 2 got 81%. Overall, the large group voted for option 2 (MMs return all results) with 81% consensus.

## 4 Discussion

As noted above, DD should both generate more authentic community member opinions and empower community members to influence the creation of an intervention. The implementation of our DD process has been both an important intellectual exercise as well as a team-building exercise for our internal research team and community members. We found the process to be feasible and believe that the process met the standards of deliberator engagement, justified reasoning and employing a societal perspective. We also believe the experience greatly enhanced the development of our planned intervention. Yet, our use of DD in Nigeria required a number of adaptations including the need for a pre-deliberation among study team members. Additionally, as would be the case in any DD or community engaged data collection process, it is critical to pay attention to cultural norms and expectations. Therefore, all images and language used in materials or presentations were reviewed by Nigerian social scientists to ensure cultural appropriateness. Further, the expert testimony videos were recorded by a Nigerian voiceover artist (in English).

Our deliberation process was subject to limitations that are likely to occur when applying DD methods in Nigeria or other LMIC settings. For example, as noted above, we veered away from the gold standard DD recruitment process, which would typically include using a community-based recruitment process whereby the large population of community members would be solicited for participation subject to a recruitment rubric that focuses on diversity of participants with regards to both demographic factors and viewpoints. In our situation, as may be the case in other LMIC settings, in-country researchers felt it was necessary to include specific individuals for the purpose of building support of the intervention that would be developed from the DD process. Additionally, while we captured 8 of the 10 perspectives our team identified as important for the deliberation, we were not able to include husbands or community members not living with HIV, finding people with these perspectives difficult to recruit. These perspectives might have increased the diversity of pros and cons from the process.

Notably, the DD process is resource intensive, especially if one is seeking geographic diversity in a large country like Nigeria. Many of our deliberation participants traveled to Lagos from elsewhere in the country, which required time and financial resources. When there is a need to include specific individuals who represent a certain viewpoint, some of which may be prominent stakeholders, significant planning is required to ensure their availability to participate. Those seeking to use a DD process in LMIC settings should weigh the resources required, the political implications and adaptations needed in the specific location with the likely impact of the data collected. In the case of our study, we believe the consensus reached regarding HPV screening helped restructure our innovation in a way that is likely to increase effectiveness of the program but the resource-intensive nature could limit feasibility in some circumstances.

## Data Availability

The raw data supporting the conclusions of this article will be made available by the authors, to the extent that data can be deidentified, without undue reservation.
